# A Relational Database for the Discovery of Genes Encoding Amino Acid Biosynthetic Enzymes in Pathogenic Fungi

**DOI:** 10.1002/cfg.236

**Published:** 2003-02

**Authors:** Peter F. Giles, Darren M. Soanes, Nicholas J. Talbot

**Affiliations:** School of Biological Sciences University of Exeter Washington Singer Laboratories Perry Road Exeter EX4 4QG UK

## Abstract

Fungal phytopathogens continue to cause major economic impact, either directly, through crop losses, or due to the costs of fungicide application. Attempts to understand these organisms are hampered by a lack of fungal genome sequence data. A need exists, however, to develop specific bioinformatics tools to collate and analyse the sequence data that currently is available. A web-accessible gene discovery database (http://cogeme.ex.ac.uk/biosynthesis.html) was developed as a demonstration tool for the analysis of metabolic and signal transduction pathways in pathogenic fungi using incomplete gene inventories. Using Bayesian probability to analyse the currently available gene information from pathogenic fungi, we provide evidence that the obligate pathogen *Blumeria graminis* possesses all amino acid biosynthetic pathways found in free-living fungi, such as *Saccharomyces cerevisiae*. Phylogenetic analysis was also used to deduce a gene history of succinate-semialdehyde dehydrogenase, an enzyme in the glutamate and lysine biosynthesis pathways. The database provides a tool and methodology to researchers to direct experimentation towards predicting pathway conservation in pathogenic microorganisms.


**Comparative and Functional Genomics**
*Comp Funct Genom* 2003; **4**: 4–15.
Published online in Wiley InterScience (www.interscience.wiley.com). **DOI:** 10.1002/cfg.236

**Research Article**

### A relational database for the discovery of genes encoding amino acid biosynthetic enzymes in pathogenic fungi

Peter F. Giles, Darren M. Soanes and Nicholas J. Talbot*
*School of Biological Sciences, University of Exeter, Washington Singer Laboratories, Perry Road, Exeter EX4 4QG, UK*

**Correspondence to:*
*Nicholas J. Talbot, School of Biological Sciences, University of Exeter, Washington Singer Laboratories, Perry Road, Exeter EX4 4QG, UK.*
*E-mail: n.j.talbot@exeter.ac.uk*

Received: 3 September 2002
Accepted: 22 November 2002

### Abstract

**Fungal phytopathogens continue to cause major economic impact, either directly, through crop losses, or due to the costs of fungicide application. Attempts to understand these organisms are hampered by a lack of fungal genome sequence data. A need exists, however, to develop specific bioinformatics tools to collate and analyse the sequence data that currently is available. A web-accessible gene discovery database (http://cogeme.ex.ac.uk/biosynthesis.html) was developed as a demonstration tool for the analysis of metabolic and signal transduction pathways in pathogenic fungi using incomplete gene inventories. Using Bayesian probability to analyse the currently available gene information from pathogenic fungi, we provide evidence that the obligate pathogen *Blumeria graminis* possesses all amino acid biosynthetic pathways found in free-living fungi, such as *Saccharomyces cerevisiae*. Phylogenetic analysis was also used to deduce a gene history of succinate-semialdehyde dehydrogenase, an enzyme in the glutamate and lysine biosynthesis pathways. The database provides a tool and methodology to researchers to direct experimentation towards predicting pathway conservation in pathogenic microorganisms.** **Copyright © 2003 John Wiley & Sons, Ltd.**

**Keywords:** **phytopathogens; metabolic pathways; ESTs; pathway analysis**

### Introduction

Fungal pathogens are responsible for serious diseases of humans and plants and are of considerable economic importance (Baker *et al*., 1997). In spite of this, there are few genomic resources available for phytopathogens, a situation that extends to fungi generally. The complete genomes of only three fungi are available in the public domain: *Saccharomyces cerevisiae* (Goffeau *et al*., 1996); *Schizosaccharomyces pombe* (Wood *et al*., 2002) and *Neurospora crassa* (**http://www-genome.wi.mit.edu/annotation/fungi/neurospora/**), all of which are non-pathogenic species. The first draft of the genome sequence of a plant pathogenic fungus *Magnaporthe grisea* has very recently become available (**http://www-genome.wi.mit.edu/annotation/fungi/magnaporthe/**), along with the first draft of the sequence of a human pathogen, *Aspergillus fumigatus* (**http://www.tigr.org/tdb/e2k1/afu1/**). In the absence of a large number of completed phytopathogenic fungal genomes, single-pass, partial sequencing of either 3′ or 5′ ends of complementary DNA (cDNA) clones to generate a set of ESTs, offers a low-cost strategy to identify substantial gene inventories. EST sequences are available for a number of fungal pathogens (reviewed in Soanes *et al*., 2002). A large number of phytopathogenic fungal ESTs have been deposited with annotation in a fungal EST database generated by the Consortium for the Genomics of Microbial Eukaryotes (COGEME: **http://cogeme.ex.ac.uk**).

The application of molecular genetic analysis to the study of phytopathogenic fungi has led to the identification and characterization of a number

Copyright © 2003 John Wiley & Sons, Ltd.

of genes involved in fungal pathogenicity (Idnurm and Howlett, 2001; Knogge, 1998; Sweigard *et al*., 1998). Some of the encoded pathogenicity factors have important roles in fungal metabolism, e.g. in the control of nitrogen source utilisation (Lau and Hamer, 1996) or the biosynthesis of amino acids (Balhadère *et al*., 1999). During the invasion of plant tissue, fungal metabolism needs to be specifically adapted to make use of the resources available from living host material. An understanding of these metabolic pathways is therefore of interest, because they offer new targets for development of novel fungicides, e.g. methionine biosynthesis has already been indicated to be the target of the anilinopyrimidine class of fungicides (Fritz *et al*., 1997).

Although the gene inventories contained in EST datasets represent only a fraction of the transcriptome, careful analysis of the genes represented in these collections can give valuable information about metabolic pathways that are present in different species of phytopathogenic fungi. Comparison of this data with that available from saprophytic fungi may provide insight into the nature of pathogenesis. Here, we present an amino acid biosynthesis gene discovery database that has been developed as a demonstration tool to show how pathway information can be gained from incomplete EST data. Amino acid biosynthesis pathways are reasonably well understood and documented (Braus, 1991; Thomas and Surdin-Kerjan, 1997). A relational database, with a web interface offering analytic functions, has been developed to exploit the available fungal genomic data with a view to investigating the conservation of amino acid biosynthesis pathways of several pathogenic species. Using the database we have investigated pathway conservation in a number of poorly characterized fungal pathogens and provide the first analysis of amino acid biosynthetic ability in an obligately pathogenic species.

### Materials and methods

### Data sources

Sequences from *Saccharomyces cerevisiae* encoding amino acid biosynthetic enzymes were selected from the SWISS-PROT database (**http://ca.expasy.org/sprot/**). Expressed sequence tags (ESTs) for the barley powdery mildew fungus *Blumeria graminis* f. sp. *hordei* were obtained from Dr S. J. Gurr (University of Oxford) and from the Carlsberg Institute, Denmark. ESTs were generated from cDNA libraries constructed from infected plant material or from germinated spores. Hierarchical clustering software was used to identify ESTs representing the same gene and produce a single contig or consensus sequence (denoted a 'unigene'). The dataset used comprised 2701 unigenes with an average sequence length of 496 base pairs. The sequences for the end rot pathogen *Botryotinia fuckeliana* (anamorph: *Botrytis cinerea*) comprised ESTs downloaded from the website of the French sequencing centre, Genoscope (**http://www.genoscope.cns.fr/externe/English/Projets/Resultats/rapport.html**). These were sequenced from a cDNA library created using *B. fuckeliana* grown under conditions of nitrogen deprivation. The data set used comprised 6558 sequences, with an average length of 602 base pairs. A total of 11 328 ESTs of the maize pathogen *Gibberella zeae* (with an average length of 924 base pairs) were downloaded from the personal web page of Dr Jin-Rong Xu at Purdue University (**http://www.genomics.purdue.edu/~jxu/Fgr/combined/seq/**). A total of 1587 ESTs of the rice blast fungus *M. grisea* (with an average length of 734 base pairs) were downloaded from the NCBI database and are data generated predominantly by Dr Ralph Dean at North Carolina State Biotechnology Centre (and formerly Clemson University). A set of 2273 unigene sequences from the wheat pathogen *Mycosphaerella graminicola* (with an average length of 645 base pairs) were provided by Dr John Hargreaves and Dr John Keon at IACR–Long Ashton. They were constructed from ESTs obtained by sequencing three cDNA libraries, two from mycelium grown in liquid culture and one from fungal infected plant material. The current release of the *Neurospora crassa* genome was downloaded from the *Neurospora* Sequencing Project at the Whitehead Institute/MIT Center for Genome Research (**http://www-genome.wi.mit.edu/annotation/fungi/neurospora/**). This represents 97% of the genome at 10-times coverage and excludes highly repetitive DNA and ribosomal RNA-encoding gene sequences. The data set used comprised

Copyright © 2003 John Wiley & Sons, Ltd. 

1705 contigs in 368 supercontigs with an average length of 22 431 base pairs. *Schizosaccharomyces pombe* genomic DNA sequences were obtained from the *Sz. pombe* Genome Sequencing Project at the Sanger Centre, UK (**http://www.sanger.ac.uk/Projects/S_pombe/**). The dataset used comprised 534 sequences with an average length of 24 608 base pairs. BLAST searches were performed against *Phytophthora infestans* (the potato late blight pathogen) ESTs at the website of the *Phytophthora* Genome Consortium Database at the National Center for Genome Resources (**http://www.ncgr.org/pgc/index.html**). Out of the 2000 EST sequences, 36 were downloaded for entry into the database. In each case where EST sequences had been previously submitted to the dbEST database, the accession numbers and records were linked to the relational database.

### BLAST

A copy of the BLAST suite of programs was downloaded from the NCBI BLAST FTP site (**ftp://ftp.ncbi.nih.gov/blast/**) for local use (Altschul *et al.*, 1990, 1997). Local BLAST databases were created from the nucleotide data sets for each species using the provided program formatdb. BLAST reports were produced locally using version 2.1.3 of TBLASTN.

### Database construction

The database was implemented using MySQL version 3.23.28 for Solaris 2.7, obtained from **http://www.mysql.com**. Figure 1A shows a schematic of the database. The database was populated using Perl scripts written using ActiveState Perl Version 5.6 for Windows (downloaded from **http://www.activestate.com**) and the Perl modules DBI, DBD-MySQL and BPlite (the latter obtained from **http://www.bioperl.org**).

### Analysis

The normalized weighted percentage identity is calculated using the expression:

$$\frac{1}{\sum_{s} l} \sum_{s} il$$

where $i$ is the percentage identity, $l$ is the length of the sequence and $s$ is the data set of sequences.

The probability of seeing a given number of enzymes in the sample set by chance is given by the expression:

$$\binom{n}{g} \frac{G!}{(G-g)!} \frac{(N-G)!}{(N-G-n+g)!} \frac{(N-n)!}{N!}$$

where $N$ is the number of genes in the genome (assumed to be 10 000), $G$ is the number of enzymes in the pathway, $n$ is the number of genes in the sample dataset and $g$ is the number of pathway enzymes in the sample data set.

### Web interface

The web interface is hosted on a Sun Ultra 10 workstation running Apache 2.0.36, and is accessible at **http://cogeme.ex.ac.uk/biosynthesis.html**. It consists of two handwritten HTML pages and 13 CGI-Perl scripts. A screenshot from this is shown in Figure 1B.

### Phylogenetic analysis

Phylip version 3.5 (**http://evolution.genetics.washington.edu/phylip.html**) was used for the production of phylogenetic trees. Four algorithms were applied to each set of data and a consensus tree derived. The four algorithms were Fitch–Margoliash, Kitsch, DNA parsimony and DNA parsimony with consensus. GeneTree version 1.1.1 (**http://taxonomy.zoology.gla.ac.uk/rod/genetree/genetree.html**) was used for the production of reconciled trees.

### Results

A relational database was produced to facilitate comparative analysis of genes encoding amino acid biosynthetic enzymes from fungal pathogen EST collections and to make the results generally accessible via the Internet. A schematic design of the database and a screen shot from the web front-end is shown in Figure 1. This can be accessed at **http://cogeme.ex.ac.uk/biosynthesis.html**. The design is such that all original information that goes into the analysis can be recovered. This includes saving the individual BLAST reports to a single

Copyright © 2003 John Wiley & Sons, Ltd.

**Figure 1.** (A) Schematic representation of the Amino Acid Gene Discovery Database. Open rectangles represent the tables in the database, names indicating the type of data stored therein. Links indicate the relationships between the tables, all links being one-to-many, the filled circle indicating 'many'. Thus, one source may be linked to many sequences and many BLAST hits. (B) Screen shot from the web-front end of the database. Species abbreviations: Blu, *Blumeria graminis*; Bot, *Botryotinia fuckeliana*; Fus, *Gibberella zeae*; Mag, *Magnaporthe grisea*; Myc, *Mycosphaerella graminicola*; Neu, *Neurospora crassa*; Phy, *Phytophthora infestans*; Sch, *Schizosaccharomyces pombe*

Copyright © 2003 John Wiley & Sons, Ltd.

table. Special-to-purpose programs were written to populate the database. A BLAST parser was used to extract the expectation values from matches in the BLAST report for use as a selection criterion for determination of pathway conservation.

The Kyoto Encyclopaedia of Genes and Genomes (**http://www.genome.ad.jp/kegg/**) was first used to identify enzymes from the amino acid biosynthesis pathways of *Saccharomyces cerevisiae*, which was used as a reference organism. A total of 75 enzymes were identified, each of which was then located in the enzyme database (**http://ca.expasy.org/enzyme/**). This led directly to one or more SWISS-PROT entries for *S. cerevisiae* from which nucleotide and translated amino acid sequences were obtained. This process yielded 93 gene sequences corresponding to individual enzymes, with 18 enzymes having two variants or subclasses in the SWISS-PROT database (**http://ca.expasy.org/sprot/**). The variants were present because some amino acid biosynthetic enzyme-encoding genes have been subject to gene duplication, e.g. asparagine synthetase (Dang *et al.*, 1996), while in other cases one protein contributes to a multimeric enzyme, e.g. the anthranilate synthase complex (Prantl *et al.*, 1985). Genetic information from eight fungal species was selected for comparison against all 93 of the *S. cerevisiae* protein sequences encoding amino acid biosynthetic enzymes. Genetic information from seven fungal species and one oomycete was selected for comparison against all 93 of the *S. cerevisiae* protein sequences encoding amino acid biosynthetic enzymes. This group contained six pathogens: the barley obligate pathogen *Blumeria graminis*; the necrotrophic end rot pathogen *Botryotinia fuckeliana* (anamorph *Botrytis cinerea*); the maize pathogen *Gibberella zeae* (anamorph *Fusarium graminearum*); the rice blast pathogen *Magnaporthe grisea*; and the wheat blotch pathogen *Mycosphaerella graminicola*; as well as two saprophytic species (the red bread mould, *Neurospora crassa*; and fission yeast, *Sz. pombe*). An oomycete pathogen, *Phytophthora infestans*, which causes potato late blight disease, was also included in the analysis as an example of a distantly related microorganism that shares the ability to cause disease in plants.

BLAST searches were performed against EST or genomic DNA data from the eight fungal species listed above, using the 93 enzyme-encoding gene sequences obtained from *S. cerevisiae* in order to identify homologous sequences in each fungal species. Up to five best hits were selected, with an expectation value less than $10^{-5}$ for each enzyme in each fungal species (Anderson and Brass, 1998). For each set of hits for a particular enzyme/species pair, the normalized, weighted percentage amino acid identity was calculated. This value is equal to the sum of the products of the percentage identity and the subsequence length of the region of similarity, divided by the sum of the subsequence lengths. The value offers a single figure representing the overall quality of the similarities between EST sequences and full gene sequences from *S. cerevisiae*.

For each amino acid synthesis pathway in each fungal species, we calculated the number of enzymes present in the datasets, assuming that the presence of a hit with an expectation value of less than $10^{-5}$ indicated that a gene encoding the enzyme was present in the fungal species (Figure 1). The second value calculated was the probability that the number of enzymes identified in each pathway for each fungus could have arisen by chance. The calculation involves the number of genes in the fungal genome (assumed in each case to be 10 000), the number of EST sequences (assumed to be a random sample from the genome), the number of enzymes present in each pathway in *S. cerevisiae*, and the number of enzymes represented in the EST collection. So, for example, if there are eight enzymes present in a given pathway and an EST set contains 2500 unique sequences (one-quarter of the predicted genome), then by chance two enzymes would be expected to be present from the pathway in the EST data set (if the pathway is present in the fungal species). If the probability value calculated is less than 0.05, then the number of enzymes present in the EST collection is significantly different (at 95% confidence level) from the number expected by chance. This could be because genes encoding enzymes in the amino acid pathway are underrepresented, or overrepresented, in the EST collection. The limitation in this scheme is the assumption that EST data represents a random sample of genes in a genome, which certainly is not the case due to the dependence on expression patterns of genes at the time of cDNA library construction. Therefore, greater importance needs to be given to probability data that show the presence of an amino acid pathway rather than its

Copyright © 2003 John Wiley & Sons, Ltd.

absence. These results would indicate that a particular amino acid pathway is present in a particular species if either the probability value calculated is greater than 0.05 (i.e. the number of enzymes present in the EST data set is what we would expect to occur by chance sampling of the genome), or the probability is less than 0.05 (due to there being more genes encoding enzymes in the amino acid pathway than would be expected to occur by chance). Probability values indicating the amino acid biosynthetic pathways that are underrepresented or overrepresented in EST collections from each of the pathogenic fungi are shown in Tables 1 and 2. Since there is redundancy in enzymes fulfilling roles in more than one amino acid biosynthetic pathway, the data have been treated separately, and Table 2 shows results from enzymes that occur only in a single amino acid biosynthetic pathway. Employing this second set of data removes the potential errors rising from the presence in one pathway of an enzyme that is only active in another pathway. Of 10 significant instances in Table 1, eight arise from an unexpectedly large number of enzymes in the samples for the various pathways, suggesting that in these cases the genes encoding enzymes in these particular pathways are overrepresented in the EST data set. Two cases in Table 1 result from fewer enzymes than expected appearing in the samples. The histidine biosynthesis pathway appears to be absent from *G. zeae* based on this EST sample size, with an apparent significance at the 99.5% level. This suggests that the histidine biosynthesis pathway may be absent from this species, although consideration must be given to the nature of the data sample and the conditions under which it was produced (see discussion). Histidine biosynthetic enzymes are also underrepresented in *B. fuckeliana* (Table 1). Table 2 shows a similar split to Table 1, with the majority of instances arising from an excess of enzymes in the sample, the sole exception being *G. zeae* and the histidine biosynthesis pathway.

The most significant finding from a plant pathology standpoint, was the fact that *Blumeria graminis* appears to possess all amino acid biosynthetic pathways. *B. graminis* is an obligate parasite and

**Table 1.** Amino acid biosynthetic pathways from phytopathogenic fungi where the number of enzymes in the sample dataset, as compared to the number in the pathway of the reference species *S. cerevisiae*, differs significantly from the number expected by chance

| Fungal species | Amino acid biosynthetic pathway | Probability[1] | Sample enzymes[2] | Pathway enzymes[3] |
|---|---|---|---|---|
| *B. fuckeliana* | Histidine | 0.0312 | 1 | 8 |
| *G. zeae* | Histidine | 0.0039 | 0 | 8 |
| *M. grisea* | Glutamate | 0.0052 | 6 | 10 |
| *M. grisea* | Cysteine | 0.0279 | 4 | 7 |
| *M. graminicola* | Alanine and aspartate | 0.0222 | 7 | 12 |
| *M. graminicola* | Glutamate | 0.0290 | 6 | 10 |
| *M. graminicola* | Glycine, serine and threonine | 0.0494 | 7 | 14 |
| *M. graminicola* | Lysine | 0.0003 | 8 | 9 |
| *M. graminicola* | Methionine | 0.0162 | 6 | 9 |
| *M. graminicola* | Valine, leucine and isoleucine | 0.0009 | 7 | 8 |

[1] This is the probability that the number of enzymes from the biosynthetic pathway observed in the sample arose by chance. The cases shown are those that are significant at the 95% level, i.e. $p < 0.05$. The number of enzymes in these cases falls outside the expected range, compared to the case of *S. cerevisiae*. Note that the probabilities are numerically meaningful only if the data samples are truly random, which is unlikely. The figures should be regarded only as indicative of unexpected enzyme numbers in the samples. Note that enzyme subclasses are ignored in the calculations. An enzyme is defined as having more than one subclass if there are more than one distinct gene encoding this enzyme, either because of gene duplication or the enzyme being made up of more than one polypeptide.

[2] These are the numbers of distinct enzymes from the amino acid biosynthetic pathway for which evidence exists in the form of enzyme-encoding sequences in the sample sequence sets. By comparison with the corresponding number for the reference species in the next column, it can be determined whether there is a significant shortage or excess of enzymes in the sample. Note that enzyme subclasses are ignored in the counts.

[3] These are the numbers of distinct enzymes in the amino acid biosynthetic pathway, as determined for *S. cerevisiae* from the Kyoto Encyclopaedia of Genes and Genomes. Note that enzyme subclasses are ignored in the counts.

Copyright © 2003 John Wiley & Sons, Ltd. 

**Table 2.** Amino acid biosynthetic pathways from phytopathogenic fungi where the number of enzymes in the sample dataset, as compared to the number in the pathway of the reference species *S. cerevisiae*, differs significantly from the number expected by chance. Only enzymes that occur in a single amino acid biosynthetic pathway are considered here

| Species | Pathway | Probability[1] | Sample enzymes[2] | Pathway enzymes[3] |
|---|---|---|---|---|
| *G. zeae* | Histidine | 0.0078 | 0 | 7 |
| *B. graminis* | Methionine | 0.0430 | 4 | 5 |
| *M. grisea* | Glutamate | 0.0062 | 4 | 5 |
| *M. grisea* | Glycine, serine and threonine | 0.0447 | 4 | 8 |
| *M. graminicola* | Alanine and aspartate | 0.0500 | 4 | 6 |
| *M. graminicola* | Lysine | 0.0018 | 5 | 5 |
| *M. graminicola* | Methionine | 0.0233 | 4 | 5 |
| *M. graminicola* | Valine, leucine and isoleucine | 0.0009 | 7 | 8 |

[1] This is the probability that the number of enzymes observed in the sample and that occur only in the named biosynthetic pathway arose by chance. The cases shown are those that are significant at the 95% level, i.e. $p < 0.05$. The number of enzymes in these cases falls outside the expected range, compared to the case of *S. cerevisiae*. Note that the probabilities are numerically meaningful only if the data samples are truly random, which is unlikely. The figures should be regarded only as indicative of unexpected enzyme numbers in the samples. Note that enzyme subclasses are ignored in the calculations. An enzyme is defined as having more than one subclass if there are more than one distinct gene encoding this enzyme, either because of gene duplication or the enzyme being made up of more than one polypeptide.
[2] These are the numbers of distinct enzymes which occur only in the named amino acid biosynthetic pathway and for which evidence exists in the form of enzyme-encoding sequences in the sample sequence sets. By comparison with the corresponding number for the reference species in the next column, it can be determined whether there is a significant shortage or excess of enzymes in the sample. Note that enzyme subclasses are ignored in the counts.
[3] These are the numbers of distinct enzymes which occur only in the named amino acid biosynthetic pathway, as determined for *S. cerevisiae* from the Kyoto Encyclopaedia of Genes and Genomes. Note that enzyme subclasses are ignored in the counts.

cannot grow on defined axenic growth media (for reviews, see Giese *et al*., 1997; Tucker and Talbot, 2001). *B. graminis* thus relies entirely on living plant tissue as a source of nutrition. From this, it may be hypothesized that the amino acid biosynthetic pathways corresponding to amino acids that are plentiful in host plant leaves would be dispensable for its survival. The results shown in Figure 4 do not support this hypothesis and appear to indicate the contrary, that *B. graminis* possesses all of the amino acid biosynthesis pathways found in *S. cerevisiae*.

### An enzyme phylogeny

Analysis of our EST data showed that some enzymes were present for all species, e.g. glycine hydroxymethyltransferase (EC 2.1.2.1), from the glycine, serine and threonine biosynthesis pathways, or glutamine synthetase (EC 6.3.1.2), from the glutamate biosynthesis pathway. The database thus provides an opportunity to investigate the evolution of amino acid biosynthesis among the fungi selected. As an example of the potential of this type of database facility, we decided to investigate the phylogenetic relationships for the genes showing high similarity to the *S. cerevisiae* succinate-semialdehyde dehydrogenase (EC 1.2.1.16) gene, from the glutamate and lysine biosynthetic pathways. Several gene trees were produced using different phylogenetic algorithims and a consensus tree was determined. A species tree for the ascomycete fungi represented in the database was then taken from the taxonomy section of the NCBI website (**http://www.ncbi.nlm.nih.gov/Taxonomy/taxonomyhome.html/**) (Figure 2A), and the gene tree and the species tree were used to produce a reconciled tree, using the phylogenetic program GeneTree (Page, 1998), which represents the simplest embedding of the enzyme gene tree within the species tree. The embedding requires the postulation of gene duplications and gene losses that may have occurred during the evolutionary history of this step in glutamate biosynthesis. The resulting tree is shown in Figure 3 and exhibits three gene duplication events and a total of 13 gene losses that have putatively occurred among the species selected. One interpretation of this information is that the same enzymatic function is

Copyright © 2003 John Wiley & Sons, Ltd.

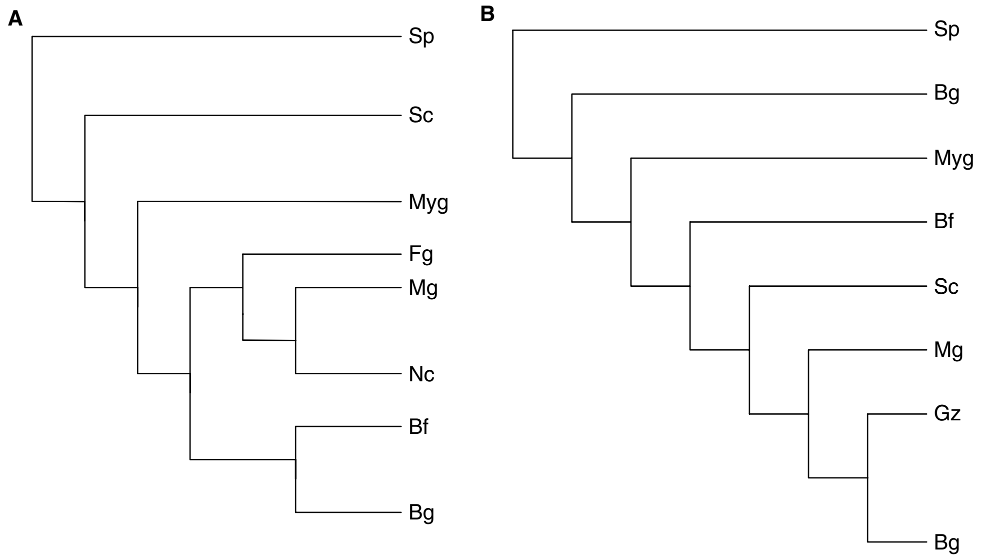

**Figure 2.** Comparison of phylogenetic relationships between the fungi used in this study. (A) The species tree drawn from NCBI taxonomy. (B) The species tree derived from phylogenetic analysis of 5.8S rRNA subunit sequences. Species abbreviations: Sc, *Saccharomyces cerevisiae*; Bg, *Blumeria graminis*; Fg, *Gibberella zeae*; Mg, *Magnaporthe grisea*; Myg, *Mycosphaerella graminicola*; Bc, *Botryotinia fuckeliana*; Pi, *Phytophthora infestans*; Nc, *Neurospora crassa*; Sp, *Schizosaccharomyces pombe*

being carried out by enzymes which are either very closely related evolutionarily, e.g. in *S. cerevisiae* and *Schizosaccharomyces pombe*, or by enzymes with quite divergent histories, e.g. *N. crassa* and *S. cerevisiae*. An alternative interpretation, which may be more likely, is that the analysis has revealed that paralogous duplication of this gene has occurred and that sequences identified in the database as succinate-semialdehyde dehydrogenase, based on identity to the *S. cerevisiae* sequence, may in fact carry out three alternative functions. If this is the case, then the gene losses indicated in the tree show the missing orthologues from each fungal species. Full genome sequence analysis of these species will ultimately reveal which of these interpretations is the most valid. This form of analysis, however, reveals the utility of collating pathway information from fungal EST sets at an early stage, in order to guide future experimentation.

In parallel with the gene phylogeny analysis, we located sequences for the 5.8S rRNA subunit for each species, a sequence comprising 158 base pairs for *S. cerevisiae*, to carry out an independent taxonomic classification of the species, in order to check the robustness of the taxonomic classification at NCBI. Originally, we had sought sequences for the 18S rRNA subunit, the sequence length being more than ten times the length of the smaller subunit. However, there was incomplete coverage of the species of interest held in the public databases. The ribosomal RNA sequences were used to provide a species tree against which the EC 1.2.1.16 tree could be compared. This provided an independent assessment of phylogenetic relationships between the species being analysed, in order to predict more accurately the likely history of the enzyme family being investigated. This tree is shown in Figure 2B. The reconciled tree resulting from this species tree and the enzyme gene tree exhibited four gene duplications and 22 gene losses (data not shown). Taken together, these results indicate the utility of using the database to examine putative evolutionary relationships among metabolic pathways in free-living and pathogenic fungi.

### Discussion

The pace of progress of fungal genome sequencing to date has been slow compared to other eukaryotes (Yoder and Turgeon, 2001; Soanes *et al.*, 2002), with the first draft genome sequence of only one phytopathogenic fungus, *Magnaponrthe grisea*

Copyright © 2003 John Wiley & Sons, Ltd.

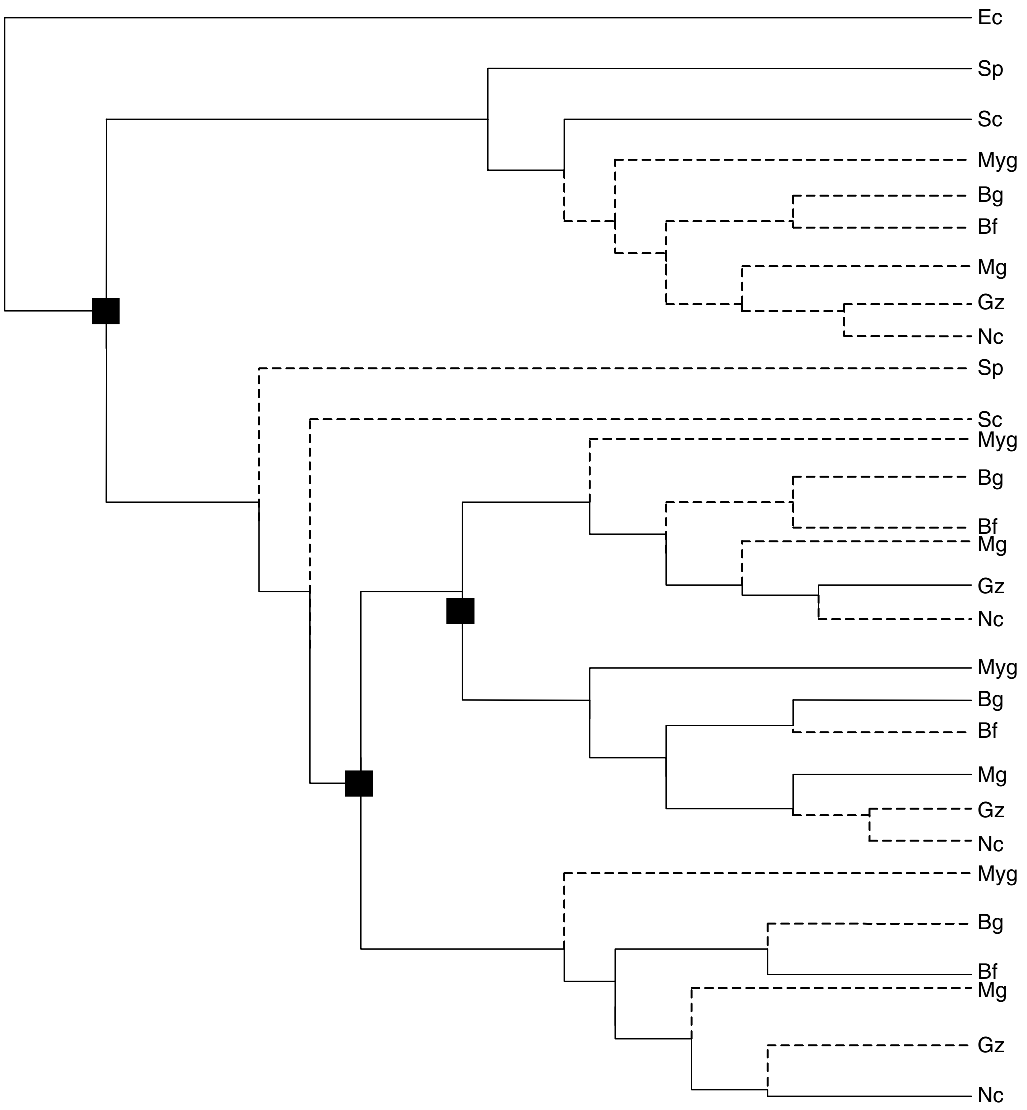

**Figure 3.** Reconciled tree for succinate-semialdehyde dehydrogenase (EC 1.2.1.16) produced from a species tree drawn from NCBI taxonomy and an enzyme tree from the best BLAST hits for each species. Black squares indicate gene duplications, dashed lines indicate gene losses. Species abbreviations: Sc, *Saccharomyces cerevisiae*; Bg, *Blumeria graminis*; Fg, *Gibberella zeae*; Mg, *Magnaporthe grisea*; Myg, *Mycosphaerella graminicola*; Bc, *Botryotinia fuckeliana*; Pi, *Phytophthora infestans*; Nc, *Neurospora crassa*; Sp, *Schizosaccharomyces pombe*; Ec, *Escherichia coli*

being publicly available (**http://www-genome.wi.mit.edu/annotation/fungi/magnaporthe/**). In the absence of complete data, it is important to extract as much useful information from the existing partial data sets as possible. To facilitate this, the development of new bioinformatic tools is required (Soanes *et al.*, 2002). An amino acid biosynthesis gene discovery database has been developed as a demonstration of the type of facility required. The relational database contains fungal sequence data drawn from a number of discrete sources. By design, the database allows comparison across fungal species or broader groupings, such as saprophytic species with pathogenic species. A Bayesian metric has been used to infer the likely presence or absence of particular amino acid biosynthetic pathways in each organism. The main limitation in this analysis results from the nature of the partial EST

data sets. These data sets are highly unlikely to meet the assumption required for validity of the probability calculations that the data is drawn randomly from the entire genome, because the abundance of mRNAs representing different genes differ at the time of cDNA library construction, depending on the growth conditions and fungal tissue used. Unigene sequence sets (used for *B. graminis* and *M. graminicola*) created by clustering EST sequences, however, have less sequence redundancy and may more closely meet the assumption on which the probability calculation is based. The remaining EST data utilized in this study, however, are from unclustered EST collections that represent the most highly expressed genes under a given set of experimental conditions (such as plant infection, starvation stress or sporulation). Notwithstanding this limitation, the probability values provide information with the potential to provide early indications of the absence or presence of a particular amino acid pathway in an organism of interest, e.g. the data suggested the absence of a histidine biosynthesis pathway in *G. zeae*. Although this is most likely due to the non-random nature of the EST dataset, it provides a potential avenue for experimental analysis of this species. The *G. zeae* ESTs used in our analysis were from four cDNA libraries derived from mature perithecia, nitrogen-starved mycelium, carbon-starved mycelium, and mycelium growing in a rich nutrient source. Given that three out of the four libraries were generated from mycelium growing under starvation conditions, it seems likely that amino acid biosynthetic genes would be among the sequences identified in derived EST collections. It will therefore be interesting to determine whether histidine is synthesized via an alternate route in this fungus. The observation that *B. fuckeliana* also showed underrepresentation in histidine biosynthetic enzymes, provides further evidence that this pathway may be worthy of experimental analysis in phytopathogens.

The predicted conservation of amino acid biosynthesis pathways in the Barley powdery mildew fungus *Blumeria graminis* was also a surprising result of our analysis, since *B. graminis* can only grow on its host plant. This fungus is an obligate pathogen that cannot be cultured on defined media (Kobayashi *et al.*, 1991); hence, the requirement for amino acid biosynthesis might be predicted to be dispensable. The data presented here suggests that this is not the case and the organism possesses amino acid biosynthetic genes in the same way as a free-living saprotroph or a facultative pathogen (Figure 4). The inability of *B. graminis* to grow on defined media may, however, be due at least in part to the absence of other biosynthetic pathways, e.g. it has been suggested that the fungus does not have enzymes for *de novo* purine synthesis (Holloman, 1984). Bioinformatic analysis of pathway conservation may therefore provide a new and important tool in determining the nature of obligate parasitism in *B. graminis*.

As some enzymes were well represented in the EST collections, the database was suitable for the analysis of phylogenetic relationships between gene sequences encoding these particular enzymes, producing a gene tree, which can be compared with a species tree, so that a predicted evolutionary history for a given gene can be inferred. Disparities arise between gene trees and species trees because of processes such as gene duplication, gene loss and lineage sorting. Reconciled trees provide a hypothetical reconstruction of the history of a gene with respect to a species. Such an analysis quantifies the relationship between the two sorts of trees in terms of a cost. The cost of a tree is expressed as the number of gene duplications and gene losses required to reconcile the gene tree to the species tree (Page and Charleston, 1997). The more common application of reconciled trees is determining an unknown species tree from available sequence data. However, where the species tree is known, this can be fixed and the reconciled tree becomes a gene history tree. Figure 3 shows a reconciled tree showing a gene history that involves three duplications and 13 losses for the succinate-semialdehyde dehydrogenase (EC 1.2.1.16) gene. Since the EST data used represents at most 20% of the genes in the entire genome, the likelihood of 'lost' genes being found in the future is high. Our relational database provides a methodology that allows analysis of this kind to be readily facilitated. Gene duplications give rise to paralogous genes, which result in proteins that perform different but related functions. In Figure 3, *B. graminis, M. grisea* and *M. graminicola* have orthologous genes, all of which are paralogues of the gene in *G. zeae*. The gene history predicted for this gene indicates either that the same orthologous enzymatic function (EC 1.2.1.16) is being carried out by enzymes with distinct evolutionary histories or, alternatively, that the EST sequences analysed

Copyright © 2003 John Wiley & Sons, Ltd.

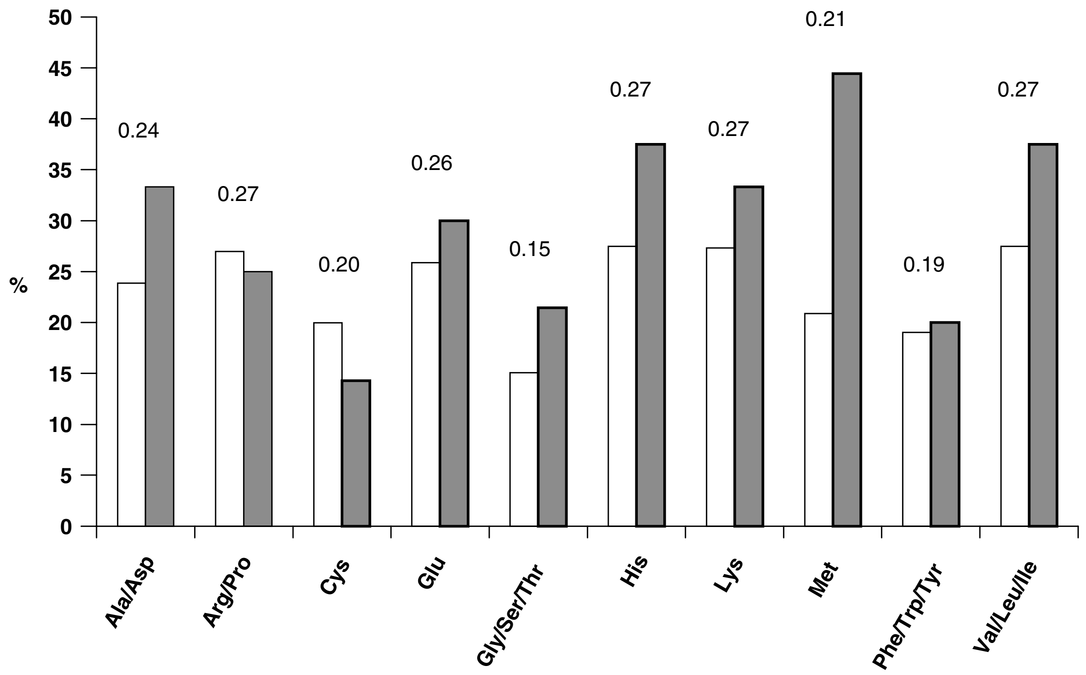

**Figure 4.** Bar chart showing the probability values for the observed numbers of amino acid biosynthetic enzyme sequences in the *Blumeria graminis* EST data set occurring by chance. The horizontal axis shows the amino acid biosynthesis pathways, arranged in 10 groups following the manner of the KEGG website. The grey bars represent the number of enzymes detected in the *B. graminis* EST data set as a percentage of the total number of enzymes in the amino acid biosynthesis pathway from *S. cerevisiae*. The white bars represent the probabilities, expressed as percentages, of observing the number of enzymes found in the *B. graminis* sequences by chance. The probability values are also indicated above the bars for each amino acid pathway represent three closely related enzymes that have evolved by paralogous duplications and may in fact be fulfilling distinct functions in different fungal species. If the latter case is true, then orthologous genes are missing from several of the EST collections. A combination of full genome sequence data and specific biochemical analysis of this enzymatic activity will be needed to reconcile this issue.

In this study, we have shown that even with the limited sequence data available for phytopathogenic fungi, collating EST data associated with a given cellular function as a single resource can provide indications of potentially significant results and guide experimental strategies. It must be stressed that these are indications only, and the need for empirical analysis remains as important as ever. However, in the absence of complete genome sequence information, our database and its schema may be used to guide the direction of experiments to investigate amino acid biosynthesis in pathogenic fungi. The pathway prediction tool is adaptable and may be of broader utility in determining the presence of other metabolic or signal transduction pathways in organisms for which only partial gene inventories are available.

### Acknowledgements

The work was supported by a grant from the Biotechnology and Biological Sciences Research Council, as part of the Consortium for Functional Genomics of Microbial Eukaryotes (COGEME).

### References

Active state: **http://www.activestate.com**.

Altschul S, Gish W, Miller W, Myers EW, Lipman DJ. 1990. Basic local alignment search tool. *J Mol Biol* **215**: 403–410.

Altschul S, Madden TL, Schaffer AA, *et al.* 1997. Gapped BLAST and PSI-BLAST: a new generation of protein database search programs. *Nucleic Acids Res* **25**: 3389–3402.

Anderson I, Brass A. 1998. Searching DNA databases for similarities to DNA sequences: when is a match significant? *Bioinformatics* **14**: 349–356.

Copyright © 2003 John Wiley & Sons, Ltd. 

Amino acid pathways database: **http://cogeme.ex.ac.uk/biosynthesis.html**.

*Aspergillus fumigatus* Genome Database: **http://www.tigr.org/tdb/e2k1/afu1/**.

Baker B, Zambryski P, Staskawicz B, Dinesh-Kumar SP. 1997. Signalling in plant-microbe interactions. *Science* **276**: 726–733.

Balhadère PV, Foster AJ, Talbot NJ. 1999. Identification of pathogenicity mutants of the rice blast fungus *Magnaporthe grisea* by insertional mutagenesis. *Mol Plant-Microbe Interact* **12**: 129–142.

Bioperl: **http://www.bioperl.org**.

Braus GH. 1991. Aromatic amino acid biosynthesis in the yeast *Saccharomyces cerevisiae*: a model system for the regulation of a eukaryotic biosynthetic pathway. *Microbiol Rev* **55**: 349–370.

COGEME fungal EST database: **http://cogeme.ex.ac.uk**.

Enzyme nomenclature database: **http://ca.expasy.org/enzyme**.

Dang VD, Valens M, Bolotin-Fukuhara M, Daignan-Fornier B. 1996. Cloning of the *ASN1* and *ASN2* genes encoding asparagine synthetases in *Saccharomyces cerevisiae*: differential regulation by the CCAAT-box-binding factor. *Mol Microbiol* **22**: 681–692.

Fritz R, Lanen C, Colas V, Leroux P. 1997. Inhibition of methionine biosynthesis in *Botrytis cinerea* by the anilinopyrimidine fungicide pyrimethanil. *Pestic Sci* **49**: 40–46.

GeneTree: **http://taxonomy.zoology.gla.ac.uk/rod/genetree/genetree.html**.

Genoscope sequence data: **http://www.genoscope.cns.fr/externe/English/Projets/Resultats/rapport.html**.

*Giberella zeae* EST sequences: **http://www.genomics.purdue.edu/~jxu/Fgr/combined/seq/**.

Giese H, Hippe-Sanwald S, Somerville S, Weller J. 1997. *Erisyphe graminis*. In *The Mycota*, vol V, part B, Carroll GC, Tudzynski P (eds). Springer-Verlag: Berlin; 55–78.

Goffeau A, Barrell BG, Bussey H, *et al*. 1996. Life with 6000 genes. *Science* **274**: 546, 563–567.

Holloman DW. 1984. Antifungal activity of substituted 2-aminopyrimidines. In *Mode of Action of Antifungal Agents*, Trinci APJ, Ryley JF (eds). Cambridge University Press: New York; 185–206.

Idnurm A, Howlett BJ. 2001. Pathogenicity genes of phytopathogenic fungi. *Mol Plant Pathol* **2**: 241–255.

Knogge W. 1998. Fungal pathogenicity. *Curr Opin Plant Biol* **1**: 324–328.

Kyoto Encyclopaedia of Genes and Genomes: **http://www.genome.ad.jp/kegg/**.

Kobayashi I, Tanaka C, Yamaoka N, Kunoj H. 1991. Morphogenesis of *Erisyphe graminis* conidia on artificial membranes. *Trans Mycol Soc Jpn* **32**: 187–198.

Lau G, Hamer JE. 1996. Regulatory genes controlling *MPG1* expression and pathogenicity in the rice blast fungus *Magnaporthe grisea*. *Plant Cell* **8**: 771–781.

*Magnaporthe grisea* database: **http://www-genome.wi.mit.edu/annotation/fungi/magnaporthe/**.

MySQL: **http://www.mysql.com**.

NCBI BLAST FTP site: **ftp://ftp.ncbi.nih.gov/blast/**.

NCBI taxonomy homepage: **http://www.ncbi.nlm.nih.gov/Taxonomy/taxonomyhome.html**.

*Neurospora crassa* database: **http://www-genome.wi.mit.edu/annotation/fungi/neurospora/**.

Page RDM. 1998. GeneTree: comparing genes and species phylogenies using reconciled trees. *Bioinformatics* **14**: 819–820.

Page RDM, Charleston MA. 1997. From gene to organismal phylogeny: reconciled trees and the gene tree/species tree problem. *Mol Phylogenet Evol* **7**: 231–240.

PHYLIP home page: **http://evolution.genetics.washington.edu/phylip.html**.

*Phytophthora* Genome Consortium: **http://www.ncgr.org/pgc/index.html**.

*Schizosaccharomyces pombe* sequencing project: **http://www.sanger.ac.uk/Projects/S_pombe**.

Prantl F, Strasser A, Aebi M, *et al*. 1985. Purification and characterization of the indole-3-glycerol phosphate synthase/anthranilate synthase complex of *Saccharomyces cerevisiae*. *Eur J Biochem* **146**: 95–100.

Soanes DM, Skinner W, Keon J, Hargreaves J, Talbot NJ. 2002. Genomics of phytopathogenic fungi and the development of bioinformatic resources. *Mol Plant-Microbe Interact* **5**: 421–427.

Sweigard JA, Carroll AM, Farrall L, Chumley FG, Valent B. 1998. *Magnaporthe grisea* pathogenicity genes obtained through insertional mutagenesis. *Mol Plant-Microbe Interact* **11**: 404–412.

SWISS-PROT: **http://ca.expasy.org/sprot/**.

Tucker SL, Talbot NJ. 2001. Surface attachment and prepenetration stage development by plant pathogenic fungi. *Ann Rev Phytopathol* **39**: 385–417.

Thomas D, Surdin-Kerjan Y. 1991. Metabolism of sulfur amino acids in *Saccharomyces cerevisiae*. *Microbiol Mol Biol Rev* **61**: 503–532.

Wood V, Gwilliam R, Rajandream MA, *et al*. 2002. The genome sequence of *Schizosaccharomyces pombe*. *Nature* **415**: 871–880.

Yoder OC, Turgeon BG. 2001. Fungal genomics and pathogenicity. *Curr Opin Plant Biol* **4**: 315–321.

Copyright © 2003 John Wiley & Sons, Ltd. 
